# Preoperative fasting and GLP-1 receptor agonists: gastric retention without increased aspiration risk

**DOI:** 10.1016/j.bjane.2026.844763

**Published:** 2026-05-01

**Authors:** Filomena Di Vezza, Marco Sanvitti, Federico Bilotta

**Affiliations:** a University of Tor Vergata, Department of Anesthesiology and Intensive Care, Rome, Italy; b “Sapienza” University of Rome, Department of Pediatric Surgery, Rome, Italy

Dear Editor,

Preoperative fasting is a cornerstone of anesthetic safety, aiming to reduce pulmonary aspiration risk by minimizing gastric volume and acidity at induction.^1^ Contemporary guidelines allow clear fluids up to 2 hours before anesthesia, reflecting a shift toward evidence-based, patient-centered fasting strategies. However, the increasing use of Glucagon-Like Peptide-1 Receptor Agonists (GLP-1RAs) has raised concerns due to their well-established effect of delaying gastric emptying, potentially leading to residual gastric contents despite guideline-compliant fasting.

Literature has consistently shown that GLP-1RAs are associated with delayed gastric emptying and increased residual gastric contents. Evidence from a large systematic review and meta-analysis shows a higher prevalence of retained gastric contents in patients receiving GLP-1RAs, particularly when administered in the days preceding surgery.^2^ These findings are supported by physiological data showing reduced antral motility, increased pyloric tone, and modulation of vagal pathways, thereby contributing to delayed gastric emptying.^3^

Despite these physiological effects, higher-quality clinical evidence does not demonstrate a corresponding increase in aspiration-related outcomes. A recent observational cohort study of more than 300.000 patients reported no significant differences in aspiration pneumonia, respiratory complications, or mortality between GLP-1RAs users and non-users.^4^ This apparent dissociation between gastric retention and aspiration risk is clinically relevant and challenges the assumption that delayed gastric emptying alone translates into increased perioperative risk.

This discrepancy may reflect advances in perioperative airway management, widespread adoption of protective strategies, and improved risk stratification, which together mitigate the clinical impact of residual gastric contents. In this context, delayed gastric emptying does not appear to reliably predict aspiration risk in modern anesthetic practice.

Multidisciplinary consensus guidance reflects this evolving evidence, recommending continuation of GLP-1RAs in most patients and emphasizing individualized perioperative risk assessment rather than routine drug discontinuation.^5^ Importantly, GLP-1RAs exert beneficial ancillary effects, including improved glycemic stability, modulation of inflammatory pathways, and potential cardiometabolic protection, which may be particularly relevant in the perioperative setting.

In conclusion, current evidence indicates that although GLP-1RAs are associated with delayed gastric emptying and increased residual gastric contents, this does not translate into a measurable increase in perioperative aspiration risk. Routine preoperative discontinuation therefore appears unwarranted. Instead, perioperative management should be guided by patient-specific risk assessment, taking into account fasting duration and, when appropriate, point-of-care gastric ultrasound, thereby avoiding unnecessary interruption of therapies with potential systemic benefits and supporting a tailored approach to the perioperative management of GLP-1RAs.

## Use of artificial intelligence

No AI or AI-assisted technologies were used in the preparation of this manuscript.

## Data availability statement

No new datasets were generated or analyzed in this study.

## PROSPERO registration

Not applicable.

## Reporting guidelines

Not applicable.

## Funding

This research received no external funding.

## Conflicts of interest

The authors declare no conflicts of interest.
